# Porcine and Human Community Reservoirs of *Enterococcus* *faecalis*, Denmark

**DOI:** 10.3201/eid1712.101584

**Published:** 2011-12

**Authors:** Jesper Larsen, Henrik C. Schønheyder, Kavindra V. Singh, Camilla H. Lester, Stefan S. Olsen, Lone J. Porsbo, Lourdes Garcia-Migura, Lars B. Jensen, Magne Bisgaard, Barbara E. Murray, Anette M. Hammerum

**Affiliations:** Statens Serum Institut, Copenhagen, Denmark (J. Larsen, C.H. Lester, S.S. Olsen, A.M. Hammerum);; Aarhus University Hospital, Aalborg, Denmark (H.C. Schønheyder);; University of Texas Medical School, Houston, TX, USA (K.V. Singh, B.E. Murray);; Technical University of Denmark, Søborg, Denmark (L.J. Porsbo);; Technical University of Denmark, Kgs. Lyngby, Denmark (L. Garcia-Migura, L.B. Jensen);; University of Copenhagen, Frederiksberg, Denmark (M. Bisgaard)

**Keywords:** Keywords: Enterococcus faecalis, endocarditis, disease reservoirs, swine, multilocus sequence typing, electrophoresis, gel, pulsed-field, biofilms, virulence factors, drug resistance, bacteria

**To the Editor:** *Enterococcus* *faecalis*, which exists commensally in the gut in warm-blooded animals and humans, is an opportunistic pathogen that causes a variety of community-acquired and health care–associated infections, such as urinary tract and intraabdominal infections, bacteremia, and endocarditis (*1*). Only a few studies have assessed the relationships between clinical *E*. *faecalis* strains; strains endemic to the health care setting; and community strains residing in humans, animals, or animal-origin food (*2*).

Recently we showed that the emergence of high-level gentamicin-resistant (HLGR) *E*. *faecalis* among patients with infective endocarditis (IE) coincided with an increase in HLGR *E*. *faecalis* in the pig population in Denmark (*3*). The majority of isolates belonged to the same clonal group (sequence type [ST] 16), suggesting that pigs constitute a community reservoir of HLGR *E*. *faecalis*. We investigated human and porcine community reservoirs of other *E*. *faecalis* clonal types associated with IE in humans in Denmark.

A total of 20 consecutive gentamicin-susceptible *E*. *faecalis* isolates were obtained from IE patients in North Denmark Region during 1996–2002 (Table A1). Cases of IE were classified as definite (n = 12) or possible (n = 8) according to the modified Duke criteria (*4*). A case of community-acquired *E*. *faecalis* infection (n = 6) was defined in accordance with **strict criteria applied for methicillin-resistant *Staphylococcus* *aureus*** (*5*); otherwise, cases were deemed to be health care associated (n = 14) (Table A1). HLGR ST16 isolates recovered from 2 IE patients during the study period have been characterized (*3*) and were excluded from the present study.

Using multilocus sequence typing (*6*), we identified 14 STs among the 20 IE isolates (Table A1), then compared them with STs from 2 collections of *E*. *faecalis* isolates collected as part of the Danish Integrated Antimicrobial Resistance Monitoring and Research Program (www.danmap.org): 1) all 14 isolates recovered from community-dwelling humans in North Denmark Region during 2002–2006 with approval from the local ethics committee ([KF] 01-006/02), which were classified into 10 STs in this study (Table A1); and 2) 19 pig isolates from 2001 that were shown in a previous study to belong to 12 STs (*7*).

Among the 14 STs identified in IE isolates, 4 (ST19, ST21, ST72, and ST306) and 2 (ST40 and ST97) were also found among isolates from community-dwelling humans and pigs, respectively (Table A1). Isolates belonging to these 6 STs were further characterized by **pulsed-field gel electrophoresis (**PFGE) by using *Sma*I and grouped into PFGE pulsotypes as described (*3*). STs and PFGE pulsotypes (A–F) were largely concordant (ST97:A, ST72:B, ST19:C, ST40:D, ST21:E, and ST306:F), except for 2 isolates belonging to ST72 and ST40, for which PFGE banding patterns (U1 and U2, respectively) were unrelated to the major PFGE pulsotypes (A–F), and 1 ST306 isolate exhibiting the ST21-like PFGE banding pattern E (Table A1).

These findings confirm the genetic relatedness of IE isolates with those from community-dwelling humans (ST72:B, ST19:C, ST21:E, and ST306:F) and pigs (ST97:A and ST40:D). Seven (64%) of 11 IE isolates belonging to these 6 clonal types originated from IE patients with health care–associated risk factors (Table A1), which suggests that health care users are predisposed to colonization and infection with *E*. *faecalis* strains residing in human and porcine community reservoirs.

Previous reports have shown that epidemiologically distinct *E*. *faecalis* populations differ in terms of biofilm formation, virulence gene content, and antimicrobial drug susceptibility profiles (*2**,**8*). Therefore, we characterized all isolates with respect to these traits. Isolates were categorized into strong, medium, weak, and nonbiofilm formers by using the method of Mohamed et al. (*8*). The presence of 12 virulence-associated and pathogenicity island genes (*ebpA*, *gelE*, ef1824, *hylA*, ef1896, ef2347, ef2505, *hylB*, *ace*, *cbh*, *esp*, and ef0571) was investigated by using colony lysates and probes that have been described elsewhere (*9*). The antimicrobial drug susceptibility profiles (ampicillin, chloramphenicol, ciprofloxacin, erythromycin, gentamicin, kanamycin, linezolid, penicillin, streptomycin, teicoplanin, tetracycline, and vancomycin) were determined by the Sensititre system (Trek Diagnostic Systems, East Grinstead, UK) in accordance with Clinical and Laboratory Standards Institute guidelines (*10*). The isolates were generally homogenous within each clonal type in terms of biofilm formation, presence of virulence-associated and pathogenicity island genes, and resistance profiles (Table A1), further supporting that IE isolates are genetically related to those from community-dwelling humans and pigs, respectively. Notably, most IE isolates were susceptible to ampicillin (100%), penicillin (100%), vancomycin (100%), high-level gentamicin (100%), and high-level streptomycin (80%), which are the drugs of choice in therapeutic regiments for *E*. *faecalis* endocarditis.

In conclusion, our results suggest that the normal intestinal microflora of humans and pigs are community reservoirs of clinical *E. faecalis* and link 2 porcine-origin clonal types of gentamicin-susceptible *E*. *faecalis*, ST97:A, and ST40:D to IE in humans in Denmark. This finding strengthens existing evidence that pigs can be a source of serious infections in humans.

## Appendix

**Table A1:** Origins and molecular and phenotypic characteristics of *Enterococcus* *faecalis* isolates*

ID (other name)	Origin†	Setting	Sampling year	MLST†	PFGE‡	Biofilm formation	Virulence-associated genes	PAI genes	Resistance profile
31438-1	IE patient	HA	1997	97	A	Weak	ebpA gelE hylA ef1896 ef2505 ace	cbh	None
130529	IE patient	HA	2000	97	A	Weak	ebpA gelE hylA ef1896 ef2505 ace	cbh	None
67190	IE patient	CA	2002	97	A	Weak	ebpA gelE hylA ef1896 ef2505 ace	cbh	None
7330616-3 (D30)	Pig	NA	2001	97	A	Weak	ebpA gelE hylA ef1896 ef2505 ace	cbh	None
28137	IE patient	HA	1996	72	B	None	ebpA gelE ef1824 hylA ef2505 hlyB ace	cbh	None
7684	IE patient	HA	1997	72	B	Weak	ebpA gelE ef1824 hylA ef2505 hlyB ace	cbh	None
33873	IE patient	HA	2002	72	B	Medium	ebpA gelE ef1824 hylA ef2505 hlyB ace	cbh	None
1293	CD human	NA	2003	72	B	Medium	ebpA gelE ef1824 hylA ef2505 hlyB ace	cbh	None
3527	CD human	NA	2006	72	B	Medium	ebpA gelE ef1824 hylA ef2505 hlyB ace	cbh	None
1745	CD human	NA	2004	72	U1	Weak	ebpA gelE ef1824 hylA ef2505 hlyB ace	cbh	TET
43674	IE patient	CA	1999	19	C	Medium	ebpA gelE ef2505 hlyB ace	None	ERY TET
2247	CD human	NA	2004	19	C	Medium	ebpA gelE ef2505 hlyB ace	cbh esp	CIP ERY TET
54869	IE patient	HA	1997	40	D	Weak	ebpA gelE hylA ef2505 hlyB ace	cbh	KAN STR TET
7330082-2 (D1)	Pig	NA	2001	40	D	Medium	ebpA gelE hylA ef2505 hlyB ace	cbh esp	None
7330321-1 (D27)	Pig	NA	2001	40	D	Medium	ebpA gelE hylA ef2505 hlyB ace	cbh esp	CHL ERY KAN STR TET
7331063-5 (D37)	Pig	NA	2001	40	D	None	ebpA gelE hylA ef2505 hlyB ace	cbh	STR
7330887-1 (D32)	Pig	NA	2001	40	U2	None	ebpA gelE hylA ef2505 hlyB ace	cbh	ERY STR
26669	IE patient	HA	1998	21	E	Weak	ebpA gelE hylA ef2505 hlyB ace	cbh	None
3162	CD human	NA	2005	21	E	Weak	ebpA gelE hylA ef2505 ace	cbh esp	TET
105049	IE patient	CA	1997	306	E	Medium	ebpA gelE hylA ef1896 ef2505 ace	cbh esp	None
127801	IE patient	CA	1999	306	F	Medium	ebpA gelE hylA ef1896 ef2505 ace	cbh esp	None
2421	CD human	NA	2004	306	F	Medium	ebpA gelE hylA ef1896 ef2505 ace	cbh esp	None
57690	IE patient	HA	2000	22	NA	Weak	ebpA gelE ef1824 hylA ef2505 hlyB ace	cbh	None
20505-1	IE patient	HA	2000	30	NA	Weak	ebpA gelE hylA ef1896 ef2505 ace	esp	TET
100087	IE patient	HA	1999	41	NA	Weak	ebpA gelE hylA ef1896 ef2505 ace	cbh esp	ERY KAN STR TET
105158	IE patient	HA	1997	55	NA	Medium	ebpA hylA ef1896 ef2505 hlyB ace	cbh esp	CHL ERY KAN STR TET
134125	IE patient	HA	2000	55	NA	Strong	ebpA hylA ef1896 ef2505 hlyB ace	cbh esp	CHL ERY KAN STR TET
29783	IE patient	HA	1999	81	NA	Medium	ebpA gelE ef1824 hylA ef2505 hlyB ace	cbh ef0571	None
120903	IE patient	CA	1999	192	NA	Medium	ebpA gelE ef1824 hylA ef2505 hlyB ace	None	TET
107137	IE patient	HA	2001	241	NA	Weak	ebpA gelE ef1824 hylA ef2505 hlyB ace	cbh ef0571	None
83232	IE patient	CA	1997	326	NA	Strong	ebpA gelE hylA ef1896 ef2505 ace	cbh esp	None
1149	CD human	NA	2003	133	NA	Strong	ebpA hylA ef1896 ef2505 ace	cbh	TET
3392	CD human	NA	2005	133	NA	Medium	ebpA hylA ef1896 ef2505 ace	cbh	None
1732	CD human	NA	2003	141	NA	None	ebpA gelE ef1824 hylA ef2505 hlyB ace	None	CIP
1028	CD human	NA	2003	168	NA	Strong	ebpA gelE ef1824 hylA ef2505 hlyB ace	cbh	None
1309	CD human	NA	2003	168	NA	Strong	ebpA gelE ef1824 hylA ef2505 hlyB ace	cbh	None
1413	CD human	NA	2003	199	NA	Medium	ebpA gelE ef1824 hylA ef2505 ace	cbh	None
2174	CD human	NA	2004	206	NA	Weak	ebpA hylA ef2505 ace	cbh	TET
2041	CD human	NA	2004	327	NA	Weak	ebpA hylA ef2505 ace	cbh	TET

*MLST, multi-locus sequence typing; PFGE, pulsed-field gel electrophoresis; PAI, pathogenicity island; IE, infective endocarditis; CD, community-dwelling; HA, healthcare-associated infection; CA, community-acquired infection; NA, not applicable; CHL, chloramphenicol; CIP, ciprofloxacin; ERY, erythromycin, KAN, kanamycin; STR, streptomycin; TET, tetracycline. †STs from 5 pig isolates (in italics) have been published previously (7); the remaining 14 pig isolates belonging to other STs (ST1, ST6, ST16, ST26, ST47, ST63, ST96, ST98, ST99, and ST100) were not included in further analysis. ‡Isolates with similar PFGE banding patterns (>82% relatedness) received the same letter designation (A–F) to reflect their genetic relatedness; highly divergent PFGE banding patterns were designated as unique (U) types (U1 and U2).
